# Intracranial Metastases Tend to Be Overt and Predict Poor Prognosis in Children With Neuroblastoma

**DOI:** 10.3389/fped.2021.716880

**Published:** 2021-11-03

**Authors:** Ying Liu, Liang Huo, Jinhua Zhang, Ying Liu

**Affiliations:** ^1^Department of Pediatrics, The Fourth Affiliated Hospital of China Medical University, Shenyang, China; ^2^Department of Pediatrics, Shengjing Hospital of China Medical University, Shenyang, China

**Keywords:** neuroblastoma, intracranial metastasis (IM), neurologic manifestation, pediatric, prognosis

## Abstract

**Background:** Neuroblastoma (NB) is the most common pediatric extracranial solid neoplasm after leukemia. Intracranial metastases (IM) rarely occur in patients with NB. The present study aimed to review the clinical characteristics of NB patients from a single center presenting with IM.

**Methods:** Two hundred children (aged 3–91 months) with NB admitted to the Fourth Affiliated Hospital of China Medical University between January 2009 and December 2015 were enrolled, and their clinical characteristics were recorded. The patients were divided into two groups based on the presence of IM. Their clinical characteristics, including demographics, clinical features, and laboratory and imaging studies, were retrospectively analyzed.

**Results:** IM occurred in 22 of 200 (11%) neuroblastoma patients, with a median age of 42.5 months (range, 3–91 months), with a male-to-female ratio of 1.4:1. Seven patients had IM at the initial diagnosis. Among the 15 children who did not have IM at initial presentation, the median interval from presentation to the diagnosis of IM was 17.3 months (range, 1–55 months). Compared with the control group, NB patients with IM tended to be asymptomatic at the time of NB diagnosis, which was made incidentally during routine physical examination (5 of 22, 22.7%, *p* < 0.05). In addition, this group had more primary intra-abdominal sites (18 of 22, 81.8%, *p* < 0.001) and worse prognosis (5 of 22, 22.7%, *p* < 0.05).

**Conclusions:** NB patients with IM have insidious onset in the early stage and a lower survival rate, especially patients with primary intra-abdominal lesions. Regular neurological monitoring could improve the rate of early diagnosis and prognosis of NB children with IM. Familiarity with the characteristic findings of NB with IM is necessary to avoid misdiagnosis and initiate necessary interventions.

## Introduction

Neuroblastoma (NB) is the most common extracranial solid malignancy that arises from neural crest cells in children (1, 2), accounting for 8–10% of all pediatric tumors and for ~15% of cancer-related deaths in children (3). The etiology of neuroblastoma remains obscure, and in most cases, there is no family history of the disease (4). NB is marked by its heterogeneous clinical behavior (5) and is classified into three risk groups (low, intermediate, and high) depending on age, extent of disease, histology, and cytogenetic abnormalities (6). The tumor may spontaneously regress or progress and metastasize with resistance to treatment (5). The incidence of NB is strongly age-dependent, with a median age of onset of 24 months and a peak age of 18 months (7). It is by far the most prevalent cancer diagnosed before the age of 1 year.

NB is an embryonal tumor of the peripheral sympathetic nervous system that typically originates from the adrenal glands, although it can develop along the paravertebral sympathetic chain (6), or anywhere along the sympathetic ganglia chain from the neck to the groin. It shows an array of incompletely understood and diverse clinical and biological characteristics and behaviors, related to the variety of locations of neuroblastic tumors and the differing degrees of histopathological differentiation (8).

Distant metastases, which frequently involve the bone, bone marrow, and liver, are present in up to 70% of children with NB at the time of diagnosis and confer a poor prognosis (9–11). Hematogenous spread to the head and neck is also commonly seen both at presentation and upon recurrence and manifests most often as osseous metastases involving the calvarium, orbit, or skull base (9). However, intracranial metastases (IM), including metastases to the brain parenchyma, leptomeninges, or cerebrospinal fluid, are rare and represent a serious complication indicating poor prognosis (12–15). Neuroblastoma with distant metastases is considered a stage 4 tumor according to the International Neuroblastoma Staging System (INSS) and has a poor prognosis (1, 16, 17).

IM in neuroblastoma patients are rare, and although some case series have focused on some specific topics such as treatment (10, 18), the role of neurosurgery (11), and imaging findings (19–21), scarce information exists on the neurologic manifestations of the disease. In this retrospective study, we aimed to describe the clinical findings of NB patients with IM in our institution. Our objective was to gain updated insights into the phenomenon of central nervous system (CNS) involvement by NB, including incidence rates, clinical features, risk factors, and survival rate. We also hoped to define the natural history of NB metastasis to the brain which may eventually lead to a better understanding of the pathophysiology of this rare disease.

## Materials and Methods

### Patient Population and Data Collection

From January 2009 to December 2015, 200 pediatric patients who were diagnosed with NB at the Fourth Affiliated Hospital of China Medical University were included and reviewed in our study. Patients with IM were defined as the IM group, while those without IM were defined as the control group. Staging was performed according to INSS. Pre-treatment risk classification was performed according to the International Neuroblastoma Risk Group system.

All NB patients were regularly followed up, and CNS imaging was deemed necessary when the patients presented with neurological symptoms, or at each half-year time point during the follow-up period. IM was confirmed by CNS imaging and was defined as all lesions located in the brain parenchyma and meninges.

The sex, age of onset, symptoms at onset, primary site of NB, time interval from symptom onset to diagnosis, metastatic sites, grade of differentiation, risk grade, N-Myc status, primary histopathology, differentiation degree, and outcome of all enrolled patients were collected and analyzed. Other clinical data, including initial neuroimaging at diagnosis, primary tumor size, histology, initial treatment, time interval from diagnosis to brain metastasis, location of IM, number of brain lesions, maximum lesion diameter, presence of edema, contrast enhancement of the lesions, type of treatment upon diagnosis of IM, neurologic outcome after treatment, concomitant extra-CNS disease at the time of IM, and survival of NB patients with IM, were reviewed. When available, autopsy reports were reviewed to determine whether the children had IM at death.

### Statistics

Statistical analyses were performed using SPSS version 22.0. Continuous data with a normal distribution were expressed as the mean ± SD and further analyzed using an independent sample *t*-test. Continuous data with abnormal distribution were analyzed using the Wilcoxon Mann–Whitney *U* test. Categorical data were expressed as absolute numbers and percentages and analyzed using the chi-square test or Fisher's exact test. Data on sex, age at onset, symptoms of onset, primary site of NB, other metastatic sites, stage, risk grade, N-Myc status, primary histopathology, differentiation degree, and outcome were analyzed using multiple logistic regression analysis. Statistical significance was set at *p* < 0.05.

## Results

### Demographic and Clinical Characteristics of NB Patients

This study included 200 patients with NB. NB was more common in boys (*n* = 125) than in girls (*n* = 75), with a male-to-female sex ratio of 1.7:1. Of the 200 children almost all (91.5%, 183/200) were older than 18 months. According to the neuroimaging results, the 200 patients were divided into the IM group (*n* = 22) and control group (*n* = 178). The demographic and clinical characteristics of the two groups are shown in Table 1. There were no statistically significant differences between the two groups regarding sex, age at first onset, symptoms of onset (fever, abdominal pain, abdominal mass, limb pain, weakness, emaciation, cough), primary site of mediastinum, combined with other metastatic sites, stage, risk grade, N-Myc status, primary histopathology, and degree of differentiation. Compared with the control group, the NB patients with IM tended to be asymptomatic at the time of NB diagnosis which was made incidentally during routine physical examination (5 of 22, 22.7% *p* < 0.05). The primary site of enterocoelia was significantly more common in the IM group than in the control group (18 of 22, 81.8% vs. 17 of 178, 9.6%, *p* < 0.001). Survival (5 of 22, 22.7% *p* < 0.05) in the IM group was significantly worse than that in the control group (82 of 178, 46.1%).

**Table 1:** Demographic and clinical characteristics of NB with and without intracranial.

	**IM group**	**Control group**	**Chi-square, *χ^2^***	***p-*value**
Sex, male	13/22	112/178	0.1226	0.7263
Age at first onset, >18 months	18/22	165/178	2.979	0.0843
Diagnosis by routine physical examination	5/22	13/178	5.687	0.0171^*^
Symptoms of onset				
Fever	10/22	46/178	3.736	0.0533
Bellyache	6/22	63/178	0.5714	0.4497
Abdominal mass	6/22	29/178	1.635	0.2010
Limb pain	8/22	36/178	2.972	0.0847
Weakness	0/22	5/178		
Become emaciated	0/22	5/178		
Cough	0/22	12/178		
Primary site				
Mediastinum	5/22	20/178	2.364	0.1242
Enterocoelia	18/22	17/178	70.83	<0.0001^****^
Combined with other metastatic sites				
Lymph node	9/22	20/178	1.703	0.1919
Bone	10/22	60/178	1.188	0.2758
Bone marrow	15/22	105/178	0.6895	0.4063
Pleural	8/22	34/178	3.517	0.0607
Spinal canal	5/22	22/178	1.802	0.1794
Lung	6/22	37/178	0.6498	0.4202
Liver	8/22	37/178	3.123	0.0772
Pancreas	0/22	5/178		
Skin	1/22	0/178		
Stage: transfer	22/22	154/178		
Risk grade: high	17/22	154/178	1.350	0.2453
NMYC (+)	12/18	45/85	1.064	0.2873
Primary histopathology	10/20	48/69	2.615	0.1059
Differentiation degree: bad	10/20	73/178	0.7724	0.4398
Prognosis/alive	5/22	82/178	4.340	0.0372^*^

*,**** *p<=0.00001*.

### Natural History of NB Patients With IM

Table 2 provides information on the natural history of the 22 patients with IM. The median age at diagnosis of NB in the IM group (range 3–91 months) was 42.5 months. The median time interval from symptom onset to NB diagnosis was 4.2 months (range 0.5–29 months). Among the 15 patients who did not have IM at initial presentation, the median time interval from NB diagnosis to IM was 17.3 months (range, 1–55 months). Ten patients (10 of 22, 45.5%) had solitary brain parenchymal metastasis. The N-Myc status of these tumors was positive in three patients and not available in 19 patients.

**Table 2:** Summary of clinical characteristics of patients at the initial diagnosis of neuroblastoma in the IM group.

**Case**	**Age/sex**	**Symptom at primary diagnosis**	**Primary site of NB**	**Histology**	**Initial imaging at NB diagnosis**	**Primary tumor size, cm**	**LP at NB diagnosis**	**TI from SOD (months)**	**MYCN amplification**	**Patient risk classification**	**Initial treatment**
1	3/F	Skull mass	Adrenal gland/bilateral	NB	Ultrasound	7.4*6.9*7.4	Not	4	NA	Intermediate	Chemotherapy
2	76/M	Fever	Adrenal gland/right	GNB	Ultrasound	3.3*2.2	Not	1	NA	High	Chemotherapy
3	48/M	Leg pain	Mediastinum/right	GNB	CT	6.4*5.8*4.0	Not	16	NA	High	Surgery + chemotherapy
4	76/F	Bellyache and leg pain	Retroperitoneal/right	GNB	Ultrasound	12*5.1*10.5	Not	2	NA	High	Surgery + chemotherapy
5	31/M	Leg pain	Retroperitoneal	NB	MRI	3.0*3.0*1.0	Not	2	NA	High	Surgery + chemotherapy
6	91/F	Leg pain	Retroperitoneal/left	GNB	CT	9.0*10.0	Not	1	NA	High	Surgery + chemotherapy+
7	15/M	Fever	Retroperitoneal	GNB	CT	6.4*5.6*2.2	Not	1.5	NA	Intermediate	Surgery
8	32/M	Incidental	Adrenal gland/left	NB	Ultrasound	6.5*3.8	Not	0.75	NA	High	Surgery + chemotherapy
9	52/F	Fever; orbital ecchymosis	Adrenal gland/right	NB	Ultrasound	8.8*8.0*7.9	Not	2	NA	High	Chemotherapy
10	88/F	Fever; pain in leg	Adrenal gland/left	ND	Ultrasound	1.5*0.8*1.0	Not	1	NA	High	Surgery + chemotherapy + SCT
11	36/M	Bellyache	Retroperitoneal	NB	CT	11.9*9.3*3.5	Not	0.5	NA	High	Surgery + chemotherapy + BMT
12	12/F	Skull mass	Retroperitoneal	NB	MRI	7.1*4.7*9.3	Not	5	NA	High	Chemotherapy
13	48/M	Fever; bellyache	Retroperitoneal/left	NB	Ultrasound	10.8*8.9*12.0	Not	9	Positive	High	Surgery + chemotherapy
14	32/M	Fever; bellyache	Posterior chest wall/right	ND	CT	2.8*1.6*1.5	Not	1	NA	High	Chemotherapy
15	19/M	Skull mass	Mediastinum	GNB	CT	8.2*1.6	Not	2	NA	High	Surgery + chemotherapy +SCT
16	30/F	Leg pain	Upper posterior mediastinum/right	ND	CT	5.4*6.1*4.8	Not	1	NA	High	Chemotherapy
17	108/M	Fever; leg pain	Adrenal gland/right	GNB	CT	9.5*7.5*5.0	Not	1	Positive	High	Surgery + chemotherapy + I^131^
18	29/M	Fever; bellyache	Retroperitoneal	NB	Ultrasound	2.6*1.4*1.5	Not	3	NA	High	Surgery + chemotherapy
19	7/F	Abdomen mass	Adrenal gland/left	NB	Ultrasound	3.0*2.5*2.0	Not	4	NA	Intermediate	Surgery + chemotherapy
20	33/M	Fever; leg pain	Retroperitoneal/left	ND	Ultrasound	7.1*4.3*10.5	Not	4	NA	High	Chemotherapy
21	41/F	Fever; bellyache; arthralgia	Adrenal gland/right	GNB	CT	2.35*3.14	Not	2	Positive	High	Surgery + chemotherapy
22	29/M	Abdominal distension	Retroperitoneal/left	NA	Ultrasound	17.4*10.3*11.4	Not	29	NA	High	Chemotherapy + surgery

*M, male; F, female; NB, neuroblastoma; GNB, ganglioneuroblastoma; ND, not-described; BMT, bone marrow transplantation; SCT, stem cell transplantation; TI, time interval; SOD, symptom onset to diagnosis*.

### Imaging at the Time of Initial NB Diagnosis

Non-contrast computed tomography scan was performed in nine patients, ultrasound scanning was the initial imaging modality of choice for 11 patients, and in two patients only contrast-enhanced magnetic resonance imaging was performed. The primary site of NB was the retroperitoneum (*n* = 10), adrenal glands (*n* = 8), and mediastinum (*n* = 3).

### Clinical Characteristics of NB Patients With IM

The most common initial signs and symptoms of brain metastases were skull mass in 11 patients, headaches in two patients, seizures in two patients, blindness, eyelid ecchymosis, and orbital ecchymosis in one patient each (Table 3). There were no symptoms of spinal cord involvement. Four of our patients were asymptomatic at the time of the IM diagnosis.

**Table 3:** Clinical characteristics of patients at the diagnosis of NB with IM.

**Case**	**Symptoms of IM**	**Location of IM**	**Time interval from Dx to IM (months)**	**No. of brain lesions**	**Maximum diameter (cm)**	**Edema/enhancement**	**LP after IM**	**Treatment after IM**	**NIAT**	**Concomitant extra-CNS disease at the time of IM**	**NSE (ng/ml) at IM**	**LDH (U/l) at BM**	**SF (ng/ml) at IM**
1	Skull mass	Skull; DM; scalp	0	8	9.2*4.8	Y/Y	N	Chemotherapy	Y	Liver; abdominal skin; lymphonodus	188.3	488.9	193
2	None	PB/Lt; subcutaneous soft tissue	15	1	3.7*4.0*2.2	N/Y	N	Chemotherapy	N	Bone marrow; lymphonodus	53.31	244.7	115.1
3	Headache	DM of FB (middle and Rt)	0	2	5.2*0.7	N/N	N	Chemotherapy +Surgery	Y	Bone; bone marrow; lymphonodus	33.96	256	1,088
4	None	FB/Bil; PB/Rt	2	3	4.1*1.2	N/N	N	Chemotherapy +Surgery	N	Bone marrow	32.8	264	295.5
5	Skull mass	Parieto-occipital juncture; nasal root	28	2	3.2*1.9	N/Y	N	Chemotherapy	N	Lung; pleura; canalis vertebralis	225.6	594	1,419
6	Skull mass	Parieto-occipital juncture/Bil; PL/Rt; DM of PL/Rt	18	4	5.1*0.9*4.9	Y/Y	Y	Chemotherapy	N	Bone	66.5	298.2	342
7	Skull mass	FTB/Lt and scalp/Lt	2	1	3.0*2.0*1.5	Y/N	N	Chemotherapy	N	Bone marrow	239.4	871.2	1,139
8	Seizure	DM of frontal, parietal, and temporal bone (Bil)	18	6	8.1*1.5*5.0	Y/Y	N	Chemotherapy	N	Bone marrow; lymphonodus.	192	611.2	1,325
9	Orbital ecchymosis	DM of PB/Bi; FB/Lt; temporal pole/Rt	0	8	10.1*1.7*5.4	Y/Y	N	Chemotherapy	Y	Bone marrow; skull	193.4	796.6	515
10	None	PB/Bil	0	2	1.7*1.3*1.5	N/N	N	Chemotherapy + Surgery +SCT	Y	None	NA	NA	NA
11	Skull mass	Parietal scalp and bone/Rt and PL/Rt	28	1	5.7*9.7*5.8	Y/Y	N	Chemotherapy	N	Bone marrow; lymphonodus	136.7	366.8	230.6
12	Skull mass; proptosis	FB/Bil; skull base	0	3	4.6*0.7	Y/Y	N	Chemotherapy	Y	None	370	890	1,150
13	Blindness	Parieto-occipital meninges/Rt; occipitotemporal meninges/Lt	36	3	3.6*0.9*3.0	Y/N	N	Chemotherapy	Y	Bone marrow; lymphonodus	370	1003.2	1,214
14	Skull mass	PL/Lt; frontal occipital meninges; orbital bone/Bil	14	6	4.5*3.0*3.8	Y/N	N	Chemotherapy	N	Bone; bone marrow	176	809	760
15	Skull mass	Temporal meninges/Lt; orbital bone/Lt; basicranial bone	0	3	6.2*0.8*4.8	Y/Y	Y	Chemotherapy +Surgery + SCT	Y	Bone; bone marrow; lymphonodus	15	256.5	720.5
16	Eyelid ecchymosis	DM of frontoparietal/Bil; DM of skull base; sellar region and clival bone	2	6	6.2*0.8*4.8	Y/Y	N	Chemotherapy	Y	Liver; pleura; bone marrow; lymphonodus	370	883.1	416.4
17	Skull mass; headache	Occipitotemporal meninges/Lt	38	2	4.5*3.0*3.8	Y/Y	Y	Chemotherapy+ II	Y	Skull; spine; ribs/Bil; pelvis; humerus/Bil	370	769.4	773.8
18	None	Occipitotemporal meninges/Lt; TB; OB	55	4	3.6*1.0*3.7	N/N	N	Chemotherapy	N	Bone; bone marrow	178.5	735	233.9
19	Skull mass	DM (FB/Bil+TB/Lt); orbital bone/Bil	20	10	3.4*1.8*2.0	Y/Y	N	Chemotherapy	Y	Skull	25.8	225.3	195.5
20	Skull mass; proptosis	Frontotemporal bone; orbital bone/Rt	0	9	4.5*3.0*2.1	Y/N	N	Chemotherapy	N	Skull; spine; orbital bone; femur/Bil; bone marrow; lymphonodus	370	1,120	2,000
21	Headache, vomiting, drowsiness	TL/Lt; + cerebellum/Lt	1	4	7.64*3.35*2.80	Y/Y	N	Chemotherapy	N	Bone; bone marrow	28.37	1,083	284
22	Seizure	MCP/Lt; Falx cerebri	22	1	1.2*1.1	Y/Y	N	Chemotherapy	N	Bone; liver	19.93	331.1	2,000

*Bil, bilateral; DM, dura mater; II, intrathecal injection; IM, intracranial metastasis; FL, frontal lobe; FTB, frontotemporal bone; FB, frontal bone; Lt, left; Rt, MCP, middle cerebellar peduncle; NIAT, neurologic improvement after treatment; OB, occipital bone; OL, occipital lobe; PB, parietal bone; PL, parietal lobe; TB, temporal bone; TL, temporal lobe; right; SF, serum ferritin*.

Brain parenchyma and cerebellum metastases were found in 8 of 200 (4%) and 2 of 200 (1%) NB patients, respectively. Meningeal metastasis was found in 12 of the 200 patients (6%).

**Figure 1:**
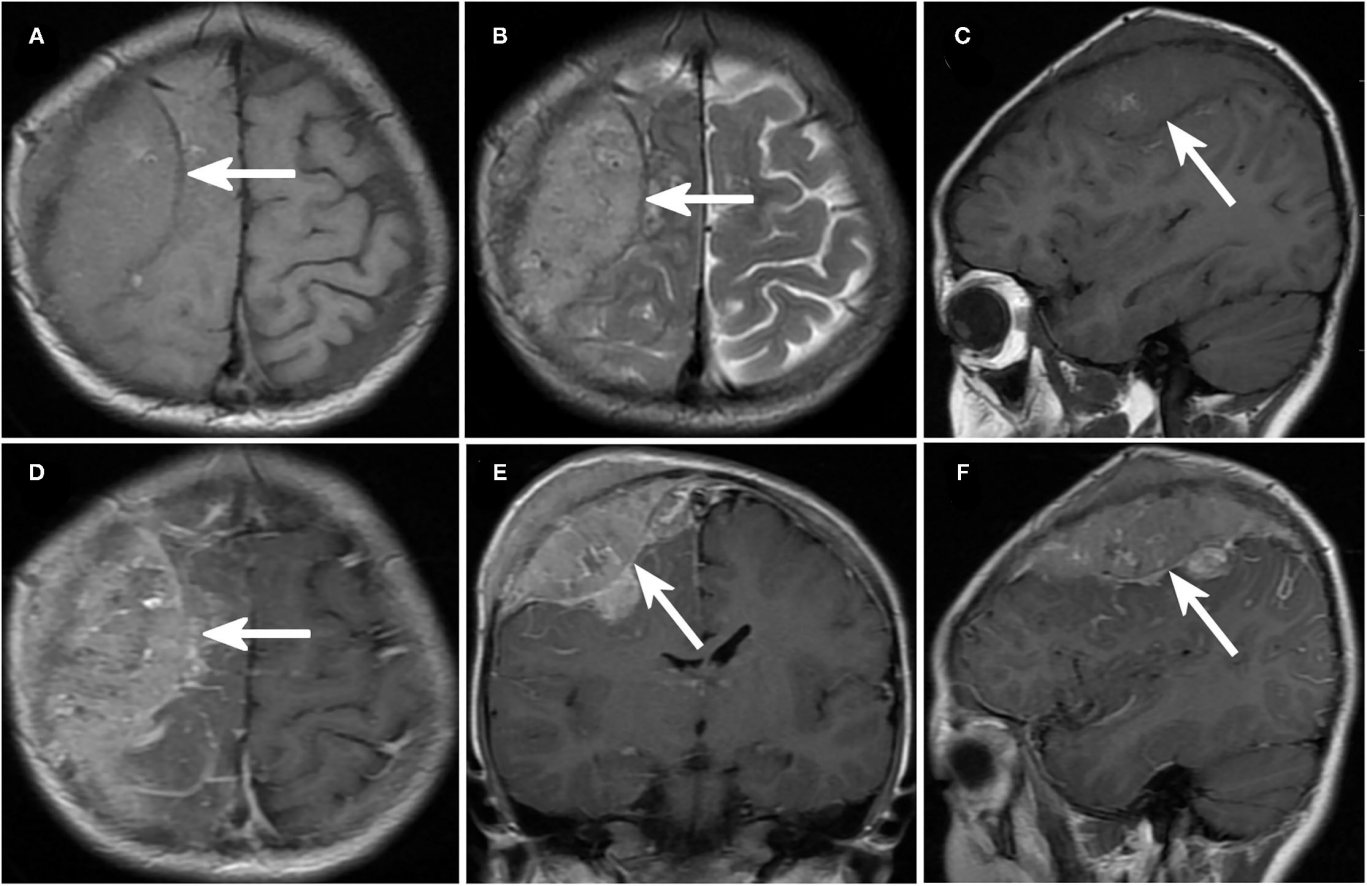
Imaging of neuroblastoma with intracranial metastases. **(A–C)** Axial **(A,B)** and sagittal **(C)** magnetic resonance imaging (MRI) in case No. 11 demonstrated a recurrent mass lesion in the right occipital lobe. **(D–F)** Cranial enhanced MRI of case No. 11, axial **(C)**, coronal (**D)**, and sagittal **(F)** images.

**Figure 2:**
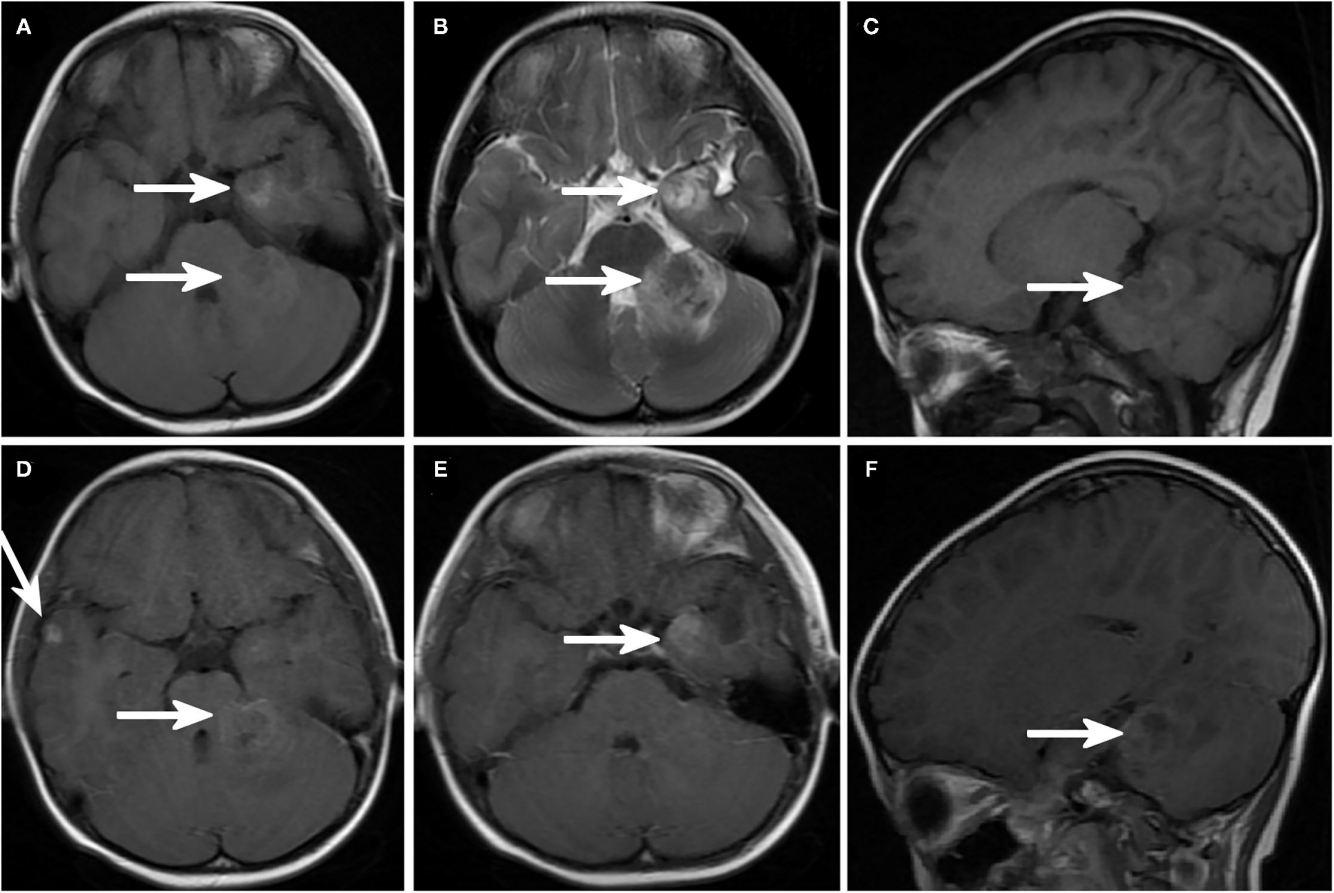
Brain magnetic resonance imaging (MRI) showing intracranial metastatic lesions. **(A)** Axial T1-weighted image and **(B)** axial T2-weighted image demonstrated a lesion in the left temporal lobe and left cerebellar hemisphere with prominent cerebral edema around the lesion. Sagittal T1-weighted image **(C)** showed a cerebellar lesion. **(D–F)** Enhanced MRI sagittal T1-weighted image and **(D)** right temporal lobe and left cerebellar hemisphere lesions. **(E)** Enhanced sagittal MRI image showed left cerebellar hemisphere lesions.

The number of brain lesions ranged from 1 to 10. In all patients with IM, brain contrast-enhanced magnetic resonance imaging was performed (Figures 1, 2). Most patients (18 of 22, 82%) had more than one mass lesion. Four patients had only one mass lesion. Most of the intraparenchymal lesions were supratentorial (19 of 22, 86%), and three lesions were infratentorial (3 of 22, 14%). The lesions in the brain parenchyma showed varying degrees of vasogenic edema in 16 patients (16 out of 22, 73%). Contrast enhancement was observed in 14 patients (14 of 22, 64%). Leptomeningeal lesions demonstrated enhancement in eight patients.

The treatment modalities for the 22 NB children with IM included chemotherapy in 17, chemotherapy plus surgery in 2, chemotherapy, surgery plus stem cell transplantation in 2, and chemotherapy plus intrathecal injection in 1. Twenty patients had concomitant extra-CNS disease at the time of brain metastases; the other common sites of extra-CNS involvement were bone (*n* = 9 patients) and bone marrow (*n* = 19 patients).

Seven patients had IM at initial presentation. Concurrent non-brain distant metastasis was present in five of these seven cases, including metastasis to the liver (*n* = 1), abdominal skin (*n* = 1), lymph node (*n* = 4), bone (*n* = 3), bone marrow (*n* = 4), and femur (*n* = 1).

By December 31, 2019, the median follow-up period had been 12.2 months (range, 1–132 months) (Table 3). During this follow-up, 17 patients had died despite treatment, four patients were alive, and one patient was lost to follow-up (Table 4). The median survival time was 12.2 months after diagnosis of IM, with a 1-year survival rate of 59.0% and 2-year survival rate of 36.4%. Of the 17 patients who died despite treatment, patient 8 died due to untreated status convulsion. Patients 13, 21, and 22 died due to multiple-organ failure, which is caused by severe infection or multiple-organ bleeding caused by bone marrow failure, cerebral herniation caused by hemorrhage of the metastatic tumor, and subarachnoid hemorrhage, respectively.

**Table 4:** Summary of prognosis of NB patients with IM.

**Case**	**Time of follow-up (months)**	**Survival time after brain metastasis (months)**	**Outcome**	**Cause**	**Age at time of death (months)**
1	102	102	Survival	NA	Survival
2	2	2	Lost to follow-up 2 m after BM	Lost to follow-up 2 m after BM	Lost to follow-up 2 m after BM
3	117	117	Survival	NA	Survival
4	26	26	Dead	NA	104
5	1	1	Dead	NA	51
6	16	16	Dead	NA	125
7	3	3	Dead	NA	20
8	2	2	Dead	Seizure	52
9	5	5	Dead	NA	49
10	31	31	Dead	NA	156
11	2	2	Dead	NA	68
12	90	90	Survival	NA	Survival
13	16	16	Dead	Osteonecrosis of bone marrow	110
14	2	2	Dead	NA	48
15	132	132	Survival	NA	Survival
16	24	24	Dead	NA	55
17	9	9	Dead	NA	156
18	17	17	Dead	NA	115
19	28	28	Dead	NA	47
20	16	16	Dead	NA	53
21	17	17	Dead	Hemorrhagic apoplexy	60
22	2	2	Dead	SAH	52

## Discussion

The objective of the present retrospective study was to describe the relevant clinical findings in NB patients with IM at our institution. We tried to gain updated insights into the phenomenon of CNS involvement by NB, including incidence rates and treatment options. To our knowledge, the present study is the largest case series to date on IM in NB patients, based on a comprehensive review of clinical features. The present study may define the natural history of IM in NB and eventually lead to a better understanding of the associated clinical characteristics with the goal of contributing to the existing knowledge of this rare disease.

NB is one of the most common extracranial solid tumors in children to metastasize to the CNS among pediatric malignant non-epithelial tumors, with an incidence of 1.7–11.7% (17, 22–24). The 22 cases reported in the present study were diagnosed with stage IV NB. Twenty-two brain metastases in a cohort of 200 neuroblastoma patients corresponds to a rate of 11%, which is close to the reported incidence in some large studies (10, 25) and higher than that reported in other studies (12–15, 17, 26). This incidence suggests that ~10% of patients will develop parenchymal brain involvement, which may justify routine CNS surveillance of this patient population. It is possible that if detected earlier, before the onset of symptoms, NB with parenchymal brain metastasis may be easier to treat.

Certain lines of evidence suggest that IM in neuroblastoma patients are rare and usually occur during disease progression (12, 13, 15, 17, 19), which was corroborated by the present study. These findings and other studies (4, 13, 27) substantiate previous indications that patients with IM have a dismal prognosis. The incidence of NB with spinal cord involvement in previous reports was 1.14–4.09% (10, 28). There was one patient (1/22, 4.5%) with involvement of the spinal cord in our study, which is consistent with previous studies.

The imaging features of IM in NB varied from single brain lesions to diffuse meningeal involvement, with a mean number of four brain lesions (range, 1–10), which is consistent with previous studies (10, 12, 27, 29, 30). Most commonly IM lesions are located in the frontal and temporal lobes (8, 31). In the 22 cases reported here, IM mainly presented as meningeal enhancement (12/22), followed by parietal lobe lesions (5/22).

It is disheartening that, by the time IM is clinically evident, it is usually too late. No consistent or effective treatment strategy exists for such patients (17, 32). The major traditional therapeutic strategies for NB are surgery, chemotherapy, radiotherapy, and autologous peripheral blood stem cell transplantation (33). Immunotherapy is an emerging potential treatment option (34, 35). In our study, chemotherapy was the mainstay treatment for NB with distant metastasis (*n* = 16). Two patients received more aggressive treatment with surgery plus chemotherapy, which may result in a longer median survival in a limited number of patients, although death from subsequent systemic recurrence seems inevitable.

In summary, IM remains an uncommon (10%) complication in children with NB. This study was based on a retrospective review of 22 cases with IM and aimed to describe the relevant clinical features of these patients in our institution. Despite being a single institution study, our report represents the largest series of children with IM described to date. Our findings lend support to the idea of routine CNS screening of children with NB to diagnose IM at earlier stages. Whether earlier diagnosis corresponds to improve survival should be the goal of prospective randomized studies.

## Data Availability Statement

The raw data supporting the conclusions of this article will be made available by the authors, without undue reservation.

## Ethics Statement

The studies involving human participants were reviewed and approved by Medical Ethics Committee of the Fourth Affiliated Hospital of China Medical University. Written informed consent to participate in this study was provided by the participants' legal guardian/next of kin. Written informed consent was obtained from the individual(s), and minor(s)' legal guardian/next of kin, for the publication of any potentially identifiable images or data included in this article.

## Author Contributions

Material preparation, data collection, and analysis were performed by YL (1st author), LH, JZ, and YL (4th author). The first draft of the manuscript was written by YL (1st author). All authors contributed to the conception and design of the study, commented on previous versions of the manuscript, and read and approved the final manuscript.

## Funding

Shenyang Science and Technology provided financial support in the form of Shenyang Science and Technology Funding (F13-221-9-43). The sponsor had no role in the design or conduct of this study.

## Conflict of Interest

The authors declare that the research was conducted in the absence of any commercial or financial relationships that could be construed as a potential conflict of interest.

## Publisher's Note

All claims expressed in this article are solely those of the authors and do not necessarily represent those of their affiliated organizations, or those of the publisher, the editors and the reviewers. Any product that may be evaluated in this article, or claim that may be made by its manufacturer, is not guaranteed or endorsed by the publisher.
